# Understanding Navon: Different designs of local-global tasks capture different bias effects

**DOI:** 10.3758/s13414-026-03300-0

**Published:** 2026-07-13

**Authors:** Felix Schweigkofler, Sjoerd Stuit, Johan Wagemans, Leendert van Maanen, Stefan van der Stigchel

**Affiliations:** 1 Utrecht University, Heidelberglaan 1, 83508 TC Utrecht, the Netherlands; 2 Katholiekee Universiteit Leuven, Tiensestraat 102 - box 3711, 3000 Leuven, Belgium

**Keywords:** Local-global processing, Global precedence, Navon task, Kimchi-Palmer task, Test-retest reliabiliy, Individual diff erences

## Abstract

When processing hierarchical compound figures with local elements in a global shape (often called the “Navon task”), an observer may perceive information from the local or the global level more quickly and/or accurately. This difference is referred to as local-global bias. This basic principle – measuring a difference between the local and global level – is conceptually very broad and can be implemented with many different task designs and figure designs. Importantly, however, there exists a pervasive notion of this bias being a unitary construct or even representative of a stable perceptual or cognitive style. However, few studies have examined the various facets of local-global bias across different task designs or assessed the retest reliability of these measures. Our study aimed to deepen the understanding of local-global bias by using three different hierarchical compound figure tasks: the Navon task and two Kimchi-Palmer task variations (one preference-based, one performance-based). We tested 75 participants across two sessions 1 week apart. Our findings revealed that metrics from the Navon task are not test-retest reliable, whereas two metrics from the Kimchi-Palmer tasks are. Some metrics were correlated between the Kimchi-Palmer tasks, but not between the Kimchi-Palmer task and the Navon task. These results suggest that local-global bias is not a unitary construct, and that different tasks measure different underlying mechanisms. We discuss possible reasons for these differences and suggest that future research should use multiple local-global tasks or otherwise prioritize Kimchi-Palmer tasks for studying individual traits and possibly Navon tasks for temporary and group-level effects.

## Introduction

### Local-global tasks

Visual information often consists of local elements that can be integrated into a global shape, traditionally described using the metaphor of trees forming a forest. Whether the local elements or global shape are perceived more quickly and/or more accurately can differ between and within individuals, and this difference is called *local-global (perception) bias*.

**Fig. 1:**
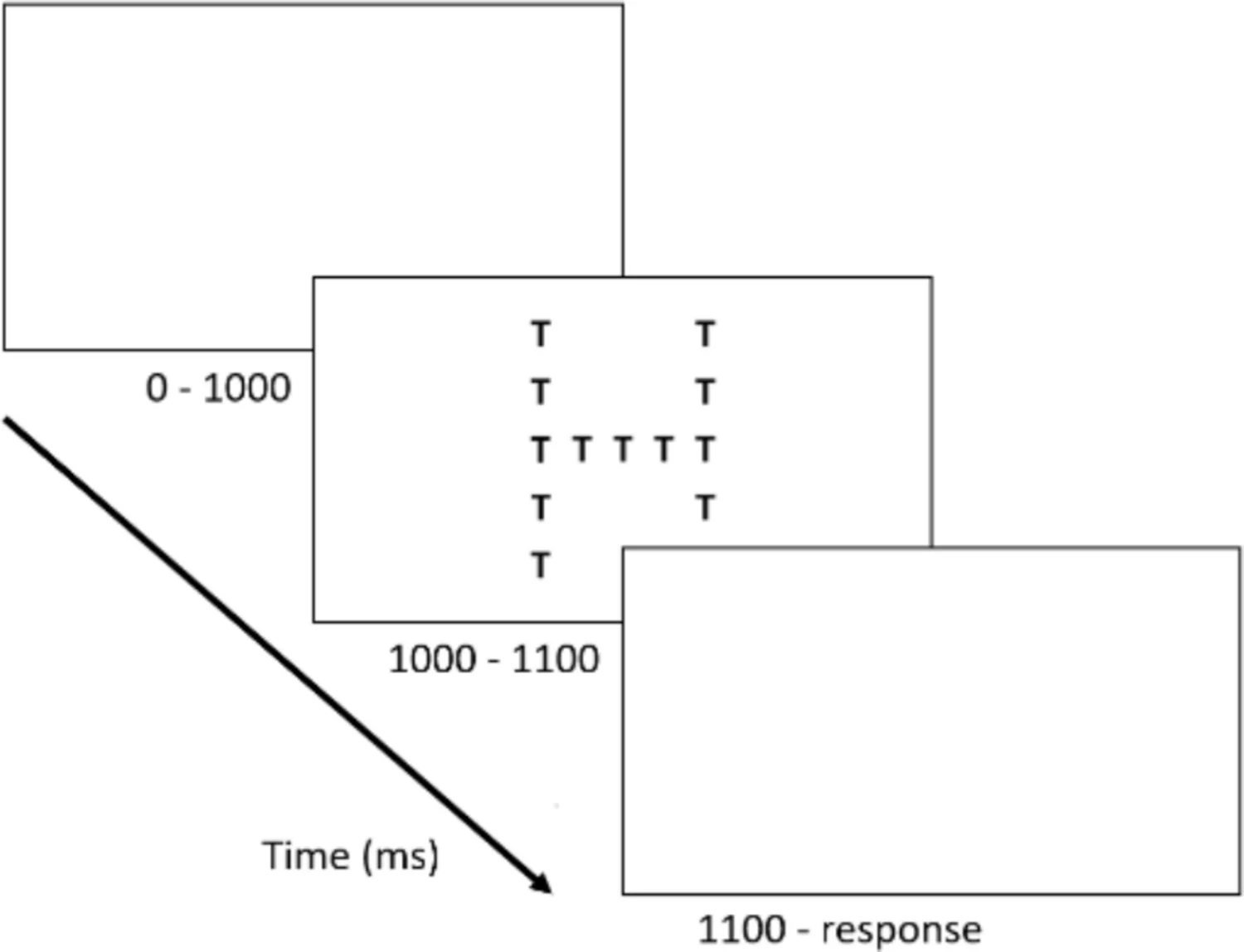
The simple Navon task. A hierarchical compound figure is shown very briefly, and the participant needs to report the feature of the target level (local or global) as quickly as possible by pressing the corresponding key on the keyboard (H or T). Usually (and in our case), the target level is defined for an entire block of trials and not for each trial individually. The figures are incongruent when local element and global shape differ, such as in this example (local T, global H), or congruent when they are the same. In the literature, the exact task design differs in many aspects, such as the size and number of the local elements, density of the local elements, familiarity of the features, number of different features in a task, and viewing duration

**Fig. 2:**
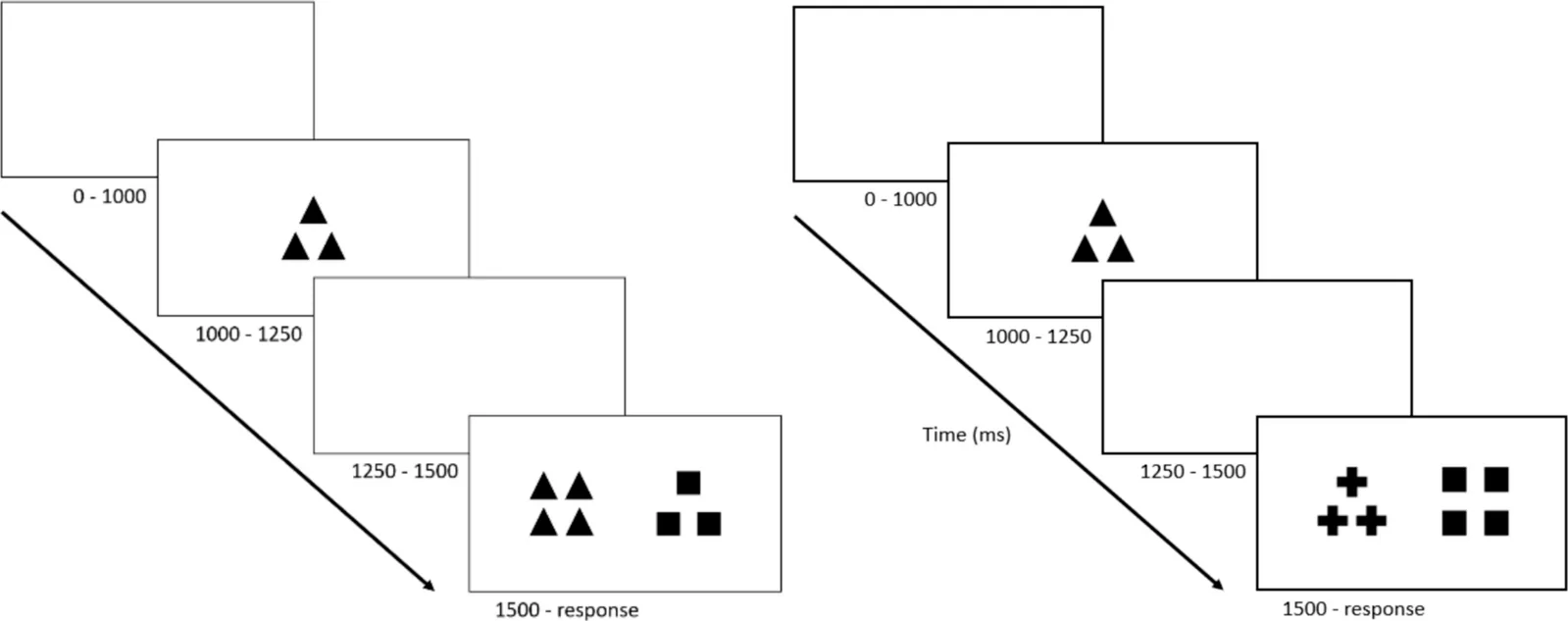
The Kimchi-Palmer task. In the preference subtask (left), the reference figure matches one of the two choice figures on the local level (here: left, triangle-elements) and one on the global level (here: right, triangle-shape). In the accuracy subtask (right), the reference figure matches only one of the two choice figures (here: left, global triangle-shape)

Participants’ local-global bias is usually estimated using hierarchical compound figure tasks, where the stimulus is a figure that is composed of local elements (e.g., triangles), forming a global shape (e.g., a square). Typical hierarchical compound figure tasks are the Kimchi-Palmer variation (Fig. 2) of the original task used by Navon (Navon, 1977) or a simplistic version of the task that is nowadays simply referred to as “the” Navon task (Fig. 1). The basic idea behind hierarchical compound figure tasks is that the two clearly distinct levels both hold different (incongruent) or identical (congruent) information, but only one of the two levels is relevant in any given trial. The task thereby captures the performance difference (bias) between the two levels.

Importantly, this concept leaves significant room for different figure and task designs. Specifically, the Navon and Kimchi-Palmer tasks used in this study differ in several key aspects.

### Task differences

Three differences between the tasks are particularly striking. Firstly, the *attentional focus* is directed towards one level in the instructions of the Navon task (directed attention), which is not the case in the Kimchi-Palmer task (divided attention). Secondly, the *complexity* is much lower in the Navon task, as every trial only uses a single figure, while each Kimchi-Palmer trial uses one reference figure to be memorized and then two figures to choose from. Thirdly, the *behavioural measures* that are obtained differ between the Navon and Kimchi-Palmer tasks, with the former generating performance measures (reaction time and accuracy) and the latter traditionally generating a preference measure (although a version generating performance measures is possible).

Due to these differences, it should not be assumed that the Navon and Kimchi-Palmer tasks are capturing identical effects. Indeed, several studies have already suggested that different hierarchical compound figure tasks are not correlated with each other (Dale & Arnell, 2013; Lacko et al., 2023; Pletzer et al., 2017). However, the notion of local-global perception bias as a unitary construct (Chamberlain et al., 2017) still persists, which seems incompatible with these non-correlations. Moreover, the evidence in the two studies reporting on the classical Navon and Kimchi-Palmer tasks did not fully consider the complexity of the local-global construct. We elaborate on this in the *Discussion* after explaining some of the complexity in the *Analysis* section in the *Methods*. To give a high-level example: Within a single local-global task, there are at least two conceptually distinct local-global bias effects (Gerlach & Krumborg, 2014), which we empirically showed to not be correlated within a simple Navon task in a meta-review using thousands of participants and slightly varying Navon figure designs (Schweigkofler et al., 2025).

### Effect sources

In the present article, we therefore attempt a more comprehensive and rigorous analysis of the local-global bias construct. For that, it is important to have an in-depth understanding of the effects that can be expected from both a “*conceptual*” and a “*functional”* point of view. For the conceptual analysis, we previously outlined in detail how the task-inherent factors *target level* (global or local) and *figure congruence* (congruent or incongruent) give rise to different effect metrics (Schweigkofler et al., 2025). Each effect metric (e.g., global condition vs. local condition in congruent trials only) has a particular conceptual meaning that we discuss in more detail in the *Analysis* section of the *Methods*. However, next to the conceptual aspect, it is important to consider the “functional” aspect: Behavioural outcome-measures in a psychological experiment are a result of the participants’ cognitive system processing external information.

The effects of interest are therefore determined by the presented information (the task design) as well as the state of the participant’s attentive-perceptual-cognitive system in that very moment. The latter is broadly speaking the sum of two components, namely the system’s stable general state (i.e., a test-retest-reliable individual trait) and temporary modulations of the system’s state (e.g., stress level). We refer to these three effect sources, the *design factor*, the *trait factor*, and the *state factor*. Trait research and state research tend to be mutually exclusive, since tasks (or questionnaires) that capture a stable trait are usually not easily influenced by temporary states and vice versa (Hedge et al., 2018). Trait and state cannot always be clearly separated, for instance, when a state is not innate but very long in duration (e.g., generalized anxiety disorder) or when a person’s susceptibility to a certain state (e.g., through priming) is in itself a trait. However, roughly separating state and trait factors is still practically useful.

The local-global literature does not usually talk about bias effects in terms of trait, state, and design explicitly, but they are implicit in the experimental design, findings, and interpretation. For example, studies have examined the impact of the design as a driver of bias and found that more densely packed local elements increase the global bias (Kimchi & Palmer, 1982). Directly comparing different tasks like the Navon and Kimchi-Palmer tasks, as we are doing, involves the design-derived bias explicitly.

Other studies have examined temporary states in relation to a participant’s (primed) mood and found that a positive mood seems to increase global bias (Fredrickson & Branigan, 2005; Gasper & Clore, 2002), while intense emotions seem to increase local bias (Noguchi & Tomoike, 2016). Using local trials or global trials to prime a participant’s cognitive state also falls into this category (Kerusauskaite et al., 2020; Klauer & Singmann, 2015), and some authors have even suggested that local-global bias effects are predominantly state driven (Förster & Dannenberg, 2010).

Trait-driven research, on the other hand, focuses on local-global bias as an innate property of a cognitive system, either by comparing individuals (Zmigrod et al., 2015) or – more frequently – by comparing different groups of interest, for example, participants with autism spectrum disorder (ASD; Ashwin et al., 2009; Lebreton et al., 2021; Mottron et al., 1999) or participants with congenital prosopagnosia, with control groups (Avidan et al., 2011; Behrmann et al., 2005). A crucial aspect of *individual* (as opposed to group-based) trait-research is the test-retest reliability of individual effects. Two studies found that several bias metrics of the Navon task do not reliably capture a participant’s bias (Dale & Arnell, 2013; Hedge et al., 2018), while one study found that one metric of the traditional Kimchi-Palmer task does (Dale & Arnell, 2013).

It was also hypothesized that the difference between the two tasks might stem from different task demands (design factor), namely that the Navon task measures a performance bias, while the classical Kimchi-Palmer task measures a preference bias (Goodhew & Edwards, 2019). To examine this hypothesis, we used a slight adaptation of the traditionally preference-based Kimchi-Palmer task to turn it into a performance-based (accuracy-based) task (see Methods).

Of all the questions that arise from interactions of the effects we described so far, we want to empirically answer two:

1. Do local-global tasks measure a stable local-global bias trait?
2. Do the Navon and Kimchi-Palmer tasks measure a unitary construct or different aspects of a complex construct?

### Unitary cognitive style

These two questions tie into the decades-old research on the existence of overarching *cognitive styles* – a term that was introduced by Witkin and refers to a certain cognitive “mode” of interacting with information (Witkin et al., 1977). Historically, local-global research stems from research on Gestalt principles and was originally designed to understand how perception works in general (Wagemans, 2015), which puts it close to the approach taken in design-driven effect research.

Since then, local-global bias seems to have been researched in relative isolation from other components of the cognitive system like visual attention (e.g., selective attention, spatial attention, focused versus distributed attention), treating it as a distinct mechanism.

However, local-global bias has also been compared between different groups with distinct perceptual-cognitive characteristics, which links it to the trait-driven research on an individual overarching cognitive style. Autism in particular has been researched extensively, with two prominent theories, the *weak central coherence* theory (Frith, 1996) and the *enhanced perceptual functioning* theory (Mottron et al., 2006), trying to explain differential bias as a function of a cognitive style. A meta-analysis has since then shown that autistic participants were slower in global trials, but not less accurate (Van der Hallen et al., 2015), demonstrating the complex nature of local-global effects.

The various theories and hypotheses regarding local-global bias have been influenced by the pervasive yet implicit notion that local-global bias is a broadly unitary construct (Chamberlain et al., 2017). If this notion does not hold up, as our (Schweigkofler et al., 2025) and others’ (Chamberlain et al., 2017; Dale & Arnell, 2013; Lacko et al., 2023; Pletzer et al., 2017) research suggests, findings may not be comparable across different studies using (possibly even just slightly) different versions of hierarchical compound figure tasks or different effect metrics. Consequently, this line of research may be based on an inaccurate understanding of the effects and therefore be hunting for shadows. For example, of 14 central coherence tasks that seem *conceptually* related, only few were found to be *empirically* related (Milne & Szczerbinski, 2009).

This pattern also seems to mostly, but notably not always, extend to the correlation between local-global bias and the vaguely related construct of field dependence (Booth, 2006; Chamberlain et al., 2017; Poirel, Pineau, Jobard, et al., 2008a, 2008b). In the Appendix (Online Supplementary Material (OSM)), we discuss this link to field dependence in more detail and report on correlations with a novel implementation of the Rod-and-Frame task, which is seen as a type of field dependence task. The focus of this article, however, lies on the local-global bias construct itself.

Treating local-global bias as a simple, unitary effect is conceptually and practically convenient, especially in the early stages of researching it. As the field is now working towards a more comprehensive understanding and integration with related frameworks, a more nuanced approach is necessary.

To support the move away from the notion of a unitary construct, we therefore previously examined and discussed within-task effect correlations (Schweigkofler et al., 2025), and we now examine across-task effect correlations, as well as test-retest reliability of different metrics and tasks. We also discuss possible implications of design-differences between the Navon and Kimchi-Palmer tasks and how to disentangle design effects in future research.

## Methods

### Participants

Seventy-five participants (28 male; age 25.4 ± 1.4 years) completed two sessions, but several participants had to be excluded (see Exclusions). Participants were recruited through the online-platform *prolific.co* with the following criteria: At least ten submissions on prolific.co, normal or corrected-to-normal vision, aged 23–28 years (incl.), UK national, fluent in English.^1^*Prolific* is a well-regarded online-platform for psychological studies (Douglas et al., 2023; Peer et al., 2021). We think our tasks are not well suited for catch-trials and we therefore rely on *Prolific’s* reputation, retest-reliabilities, and basic characteristics like error rate and reaction time to assess the quality of the data. The overall accuracy for the Navon task is high (see Appendix #1 (OSM)), as is usually reported in literature.

### Hardware and apparatus

Participants performed the browser-based, JATOS-hosted (Lange et al., 2015) experiment online on their own devices. The figure sizes (and thereby the visual angles) were kept constant, regardless of the participant’s browser size and resolution, and to achieve a constant viewing angle, participants were asked and then reminded before each task to keep a distance of 50 cm. The experiment uses the Java-Script library jsPsych 6.2.0 library (de Leeuw et al., 2023). The code to replicate the experiment can be found in the local-global bias repository (osf.io/z39j2/).

### Procedure

Participants measured the resolution of their screen by resizing a virtual box to the dimensions of a credit/library/etc. card. Then, three tasks (Kimchi-Palmer task, Navon task, and a novel implementation of the Rod-and-Frame task) were presented in this order with 1-min breaks in between to reduce fatigue. Before and after the tasks, participants were asked to report their mood (see Appendix #4 (OSM), no remarkable findings). An identical experiment was opened for the same participants a week later, with most participants completing it 7–8 days after the first session (11 days in the longest case).

### Tasks

This article focuses on the association between the Navon task and the Kimchi-Palmer task, which are hierarchical shape tasks. The Rod-and-Frame task can also be viewed from a local-global bias perspective, but it is not a hierarchical shape task and therefore falls outside of the current conceptual core of local-global theory. Furthermore, our implementation of the Rod-and-Frame task in a non-controlled online-setting is – to our knowledge – the first of its kind and not yet cross-validated with traditional implementation. For these reasons, we present our findings on the Rod-and-Frame task in the appendix (Appendix #8 (OSM)).

### Navon task

Our Navon task (Fig. 1) is based on Dale and Arnell’s design (Dale & Arnell, 2013) of the commonly used simplified version of Navon’s hierarchical compound figure task (Navon, 1977). The figures used in the task are either H or T on the global level, which are made up of either H or T on the local level, resulting in letter-congruent or letter-incongruent figures. The global shapes are displayed at 70 x 50 mm, and the local shapes are 1/10 of the size of the global shape; 70 x 50 mm appear^2^ at 8° x 5.7° visual angle for a viewing distance of 50 cm.

After a 1,000-ms blank screen, a single figure appears for 100 ms, and then the screen remains blank until a response is given. There are four blocks of 24 trials, and before each block, participants were instructed to focus on the local (first and third blocks) or global level and report the letter as quickly and accurately as possible by pressing the corresponding key. Beginning with the local block in all participants prevents any block effects from distorting the relative effects between participants and between sessions (Goodhew & Edwards, 2019).

We also analyse openly available data (osf.io/v46fa) of a fairly similar, albeit more dense, Navon design (Hedge et al., 2018).

### Kimchi-Palmer task

Our Kimchi-Palmer task (Fig. 2) is modelled closely after Dale and Arnell’s design (Dale & Arnell, 2013) of Kimchi and Palmer’s adaptation (Kimchi & Palmer, 1982) of Navon’s hierarchical compound figure task (Navon, 1977). The hierarchical compound figures used in the task are either triangles or squares on the global level, which are made up of either triangles, squares, circles, or crosses on the local level, resulting in shape-congruent or shape-incongruent figures. The global shapes are displayed at 15 x 15 mm, and two choice figures are 15 mm apart. The local shapes are 1/2.3 (triangle and cross), 1/2.56 (circle), and 1/2.76 (square) the size of the global shape; 15 mm appear at a 1.7° visual angle for a viewing distance of 50 cm.

After a 1,000-ms blank screen, a single *reference* figure appears for 250 ms, and after another 250-ms of a blank screen, two *choice* figures appear and remain on screen until a response is given. Before the trials, participants were instructed to select the choice figure that “resembles the original figure more” (with “original figure” referring to the reference figure) by pressing a key on the keyboard (without clearly stating whether the choice was free or forced). Participants were instructed not to “hurry” but still go with their “first instinct”.

In some trials, the reference figure matches one choice figure on the global and one on the local level, so that a free choice indicates a *preference* for a level (preference subtask, named KiPa-P in the figures; Fig. 2 left). In the traditionally not analysed “filler-trials”, the reference figure matches *one* choice-figure on one level (either local or global) and therefore a choice is either correct or incorrect (accuracy -ubtask, named KiPa-A in the figures; Fig. 2 right). After four practice trials without feedback, 32 P trials and 32 A trials were presented randomly. Note that preference trials use only squares and triangles as local elements, resulting in four possible combinations of reference and choice figures, each of which is used eight times. Some choice figures in accuracy trials also use crosses and circles as local elements, allowing for many more combinations (details on the chosen figure can be found in the data repository osf.io/z39j2/).

### Metrics

From the choices and reaction times gathered from these tasks, several metrics can be calculated. The most basic metric is a comparison of all local tasks with all global tasks. However, when the figure congruence is considered, a second “dimension” is added to the basic local-global dimension, giving rise to a multitude of metrics. We published on the conceptual meaning and empirical relation of these metrics in depth before (Schweigkofler et al., 2025) and showed that there are two empirically distinct bias metrics that are relevant for the Navon task, one that concerns only the bias in congruent figures, and one that describes the bias effect between the two levels interfering with each other in incongruent figures.

Critically, that analysis is based on the conceptually clear-cut single-figure Navon task. The Kimchi-Palmer task with its multiple congruent and incongruent figures per trial is more conceptually challenging than the Navon task, and in its current form we are unable to apply our metric-framework to it. For this article, we therefore fall back onto the simple *overall bias score* (OBS), which is the difference in behaviour between all local trials and all global trials (regardless of whether trials were congruent or incongruent). While the OBS is less conceptually fine-grained than the metrics we described previously, it still captures a meaningful local-global effect and is correlated with both of these more precise metrics (Schweigkofler et al., 2025).

In terms of behavioural measures, both the Navon task and the Kimchi-Palmer task generate reaction time data as well as “choice” data, i.e., which answer was given.

For the Navon task, the reaction time is commonly used and the choice data (accuracy) usually neglected. That is in part because the accuracy is very high, which leaves few errors to calculate a bias from. However, the local-global bias in accuracy is still a meaningful object of study and we therefore mention it in the text and show figures in the Appendix (OSM). From Navon we thus generate a reaction time-based OBS (rtOBS) and an accuracy-based OBS (acOBS). Note that firstly, nearly all errors are concentrated in incongruent trials (Appendix #1, Fig. S1 (OSM)), and secondly, errors in congruent trials are not directly caused by biased processing (if both levels are “H”, answering “T” cannot be assigned to the influence from the non-target level). Nevertheless, we use the accuracy *OBS* for simplicity’s sake instead of using only incongruent trials (which would result in the incongruent bias score, IBS).

For the Kimchi-Palmer task we also use both, reaction time and choice data. The traditional target metric of the task is the preference-based OBS, the participant’s bias in choosing the locally or globally matching figure when given the choice. However, this choice data in the preference subtask is also accompanied by reaction time data. Unlike in the Navon task, such a trial is not inherently local or global, but the participant’s choice can be used to code it as local or global, meaning we can generate choice data (preference) and reaction time data from this sub-task. An important caveat for this method is that strongly globally biased participants will only have a few locally coded trials and their local reaction time will therefore be based on very few trials (and vice versa for strongly locally biased participants), making the inherently noisy reaction time measure even more noisy.

Next to the preference trials, the Kimchi-Palmer task contains filler trials in which only *one* of the choice figures matches the original figure *either* on the local *or* on the global level. It is therefore – like the Navon task – a performance task in which the participant needs to quickly find the correct answer. As in the Navon task, the choice data are therefore accuracy. From this subtask we can thus also generate both, choice data and reaction time data.

We thereby obtain six metrics, three choice-based and three reaction time-based, two from Navon, and two from each Kimchi-Palmer subtask. See Table 1 for precise calculation instructions.

**Table 1:** Each Kimchi-Palmer subtask (preference, accuracy) has one reaction time-based overall bias score (OBS) and one response-based OBS (where the responses express either accuracy or preference), resulting in four different metrics. We normalize the preference OBS to a range of −1 and 1 by assigning local choices a value of −1 and global choices a value of 1 and taking their mean. We calculate all metrics such that a negative score indicates a local bias (i.e., larger reaction times mean worse performance, therefore local minus global, but large accuracies mean better performance, therefore global minus local). Only correct trials were used for calculating median reaction times

	**Navon task**	**Kimchi-Palmer task**	
		**Accuracy subtask**	**Preference subtask**
**Choice (G – L)**	Mean accuracy in global trials minus mean accuracy in local trials	Mean accuracy in global trials minus mean accuracy in local trials	Mean of local choices (coded as −1) and global choices (coded as +1), i.e., in principle global minus local values
**Reaction time** **(L – G)**	Median reaction time in local trials minus median reaction time in global trials	Median reaction time in local trials minus median reaction time in global trials	Median reaction time in trials where the participant made a local choice minus median reaction time in trials where he made a global choice

By generating data from both the preference subtask and the accuracy subtask, we can address a recent hypothesis on the observed trait stability of Kimchi-Palmer. The hypothesis states that the bias in the traditional Kimchi-Palmer task might have a higher trait stability than the bias in the Navon task, because the former traditionally examines only the preference while the latter examines performance (Goodhew & Edwards, 2019). By comparing the performance-based accuracy subtask with the traditional preference-metric, we can test whether the differential reliability is indeed due to a preference-performance dichotomy or whether it must be due to other design characteristics.

### Statistics

We use simple Pearson correlations for association statistics. To reduce the impact of outliers and give a sense of the spread of potential correlations we plot Pearson correlation coefficients from 1,000 bootstrapped samples (nonparametric bootstrap: sampling with replacement, resample size is 100% of non-excluded participants) and report their median.

To give an estimate of statistical significance, we use two-tailed Bayes factors calculated from the median Pearson’s r and the number of participants used in the correlation. Bayes factors were computed using a two-tailed Jeffreys test for correlation with a default weakly informative prior (α = 1) on the correlation coefficient (Ly et al., 2016). The benefit of Bayesian statistics over frequentist statistics is that it can provide concrete evidence for both H1 (presence of a correlation) and H0 (absence or a correlation), while frequentist statistics can only reject H0, but failure to reject it cannot be seen as statistical evidence for the absence of a correlation.

**Fig. 3:**
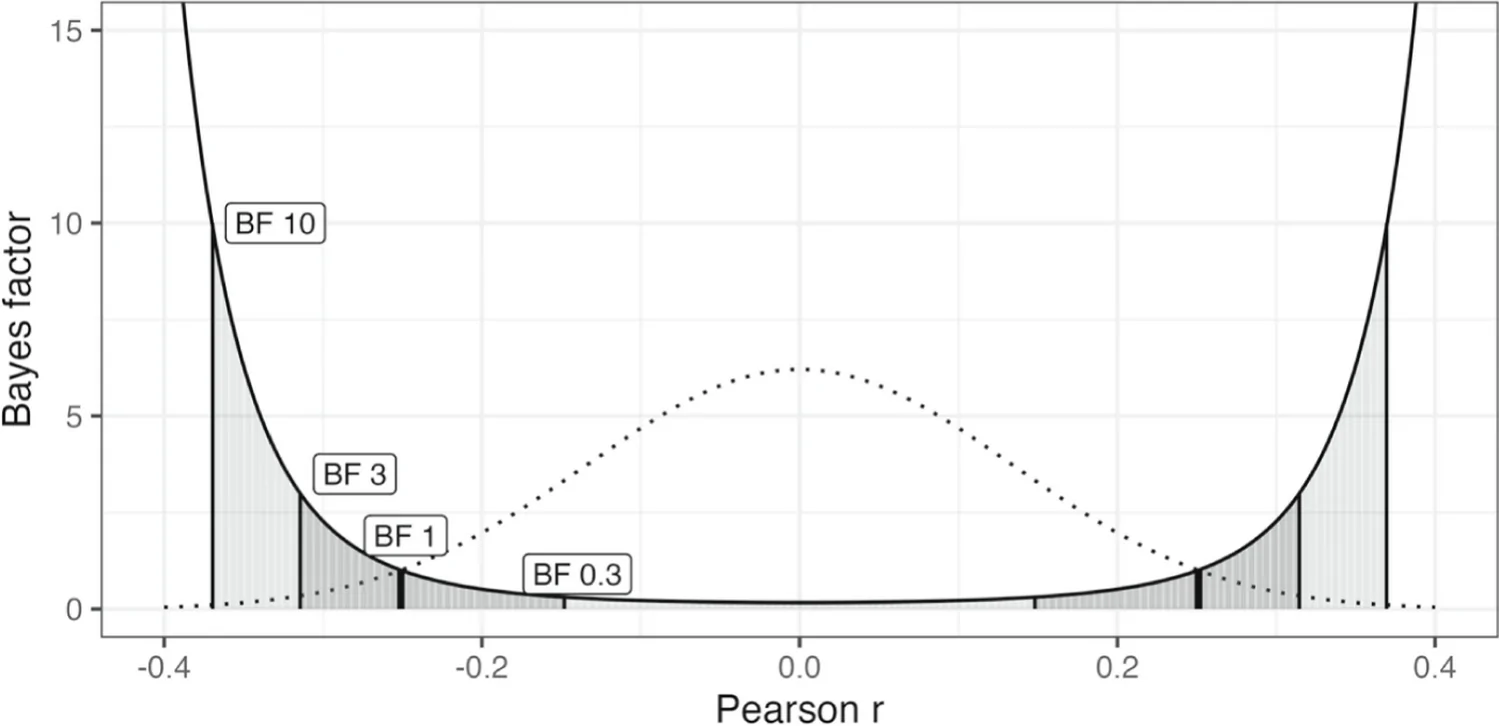
Bayes factors (BFs) for a range of Pearson’s r for an example sample size of 60 participants (which sits at the lower end of number of participants used in our correlations). BFs increase for large r and decrease for small r. Ranges of Pearson r for which there is strong evidence in favour of H1 (very left and very right) are shaded in white, ranges for which there is moderate evidence in favour of H1 (between BF 10 and BF 3) or in favour of H0 (smaller than BF 0.3) are shaded in light grey, and ranges for which there is moderate evidence in favour of H1 (between BF 3 and BF 1) or in favour of H0 (between BF 0.3 and BF 1) are shaded in dark grey. The dotted line shows 1/BF (i.e., BF 0.3 is visualized as BF 3) to give an intuitive sense of how strong the evidence in favour of H0 can be compared to evidence in favour of H1 (for correlations involving 60 participants)

Bayes factors larger than 1 indicate more evidence for H1 than for H0 and Bayes factors smaller than 1 indicate the opposite (Fig. 3). The range between 1 and 3 is considered to be anecdotal evidence for H1 and, symmetrically, the range between 1 and 0.3 is considered to be anecdotal evidence for H0 (Lee & Wagenmakers, 2013). The ranges between 3 and 10 or between 0.3 and 0.1 are considered moderate evidence for H1 and H0, respectively. The ranges beyond 10 or below 0.1 are considered strong evidence for H1 and H0, respectively.

We also visualize these ranges in the correlation plots by shading the range of Pearson r where the corresponding Bayes factors provide strong evidence in white, the range where they provide moderate evidence in light grey, and the range where they provide anecdotal evidence in dark grey (compare Fig. 3). We also note the Bayes factor values inside the plots.

Note that our number of participants is too small to reach strong evidence in favour of H0 for a single correlation, even for Pearson’s r approaching 0 (strong evidence for H0 can only be obtained with more than 150 participants; Appendix #5, Fig. S11 (OSM)). However, given that the implicit assumption of a unitary local global bias would require evidence *for* H1, we already view moderate evidence for H0 as a rejection of this assumption.

Data and analysis code (written in R) can be found on the project repository (osf.io/cm3nh/).

## Exclusions

### Participant exclusion

Three of 78 participants who completed the first session did not return for the second session and were excluded.

In the Navon task, a few participants responded in exactly the opposite way to how they should in a specific block of trials (i.e., they treated, e.g., a block of local trials as if it was a block of global trials), indicating that they missed or misunderstood or mixed up the brief instruction presented before that specific block. As these patients mostly carried out the “reversed” instructions dutifully, we see this as an issue of miscommunication and not as an indication that our web-sourced data is of low quality. See details in Appendix #1, Fig. S5 (OSM).

Participants were excluded per task per session (not from the entire study) with the following criteria:

- In the Navon task: An accuracy below 0.6 in any single trial-condition (target level × figure congruence). As a result, 14 participants were excluded in Session 1 (61 remaining) and seven in Session 2 (these include the above-mentioned misunderstanding about the instructions, 68 remaining).
- In the Kimchi-Palmer accuracy-subtask: An accuracy below 0.5 in the trials of either level (global or local). As a result, one participant was excluded in Session 1 and one in Session 2.
- In the Kimchi-Palmer preference-subtask: No participants were excluded but strongly biased participants have very little or no reaction time data for their non-preferred level. The former leads to high variance (especially for split-half correlations) and the latter leads to lower participant numbers being reported in the figures.

### Trial trimming

The median is much more robust against outliers than the mean and we therefore removed only trials that we saw as gross outliers: trials that were faster than 100 ms (unreasonably fast) or slower than 3,000 ms in the Navon task and slower than 5,000 ms in the Kimchi-Palmer task (less than 1% for each task). Only correct trials were used for calculating median reaction times (see accuracy plots in Appendix #1 (OSM)).

### Score exclusion

Some scores (OBS) are more than three times the standard deviation smaller or larger than the mean (≈0.5% of all scores, see histograms in Appendix #6 (OSM)). We did not exclude any scores.

## Results

In this study we aimed to empirically answer two main questions:

- Firstly, are the effects of two hierarchical compound figure tasks (Navon task and Kimchi-Palmer task) test-retest reliable and thus representative of an *individual trait*?
- Secondly, are the effects in the Navon and Kimchi-Palmer tasks correlated and thus representative of a *unitary local-global bias* as a perceptual style?

Note that our sample sizes between 61 and 75 are modest and that correlation values therefore are approximations. To respect this uncertainty, we discuss our correlation strengths with one decimal in the text and report them with two decimals in the figures.

Our results for group-level effects like average reaction time, error rate, and population bias are broadly in line with the literature (see Appendix #1 (OSM)), suggesting that our data are of sufficient quality and allow us to derive the relevant metrics.

### Reliability correlations

#### Split-half reliability

**Fig. 4:**
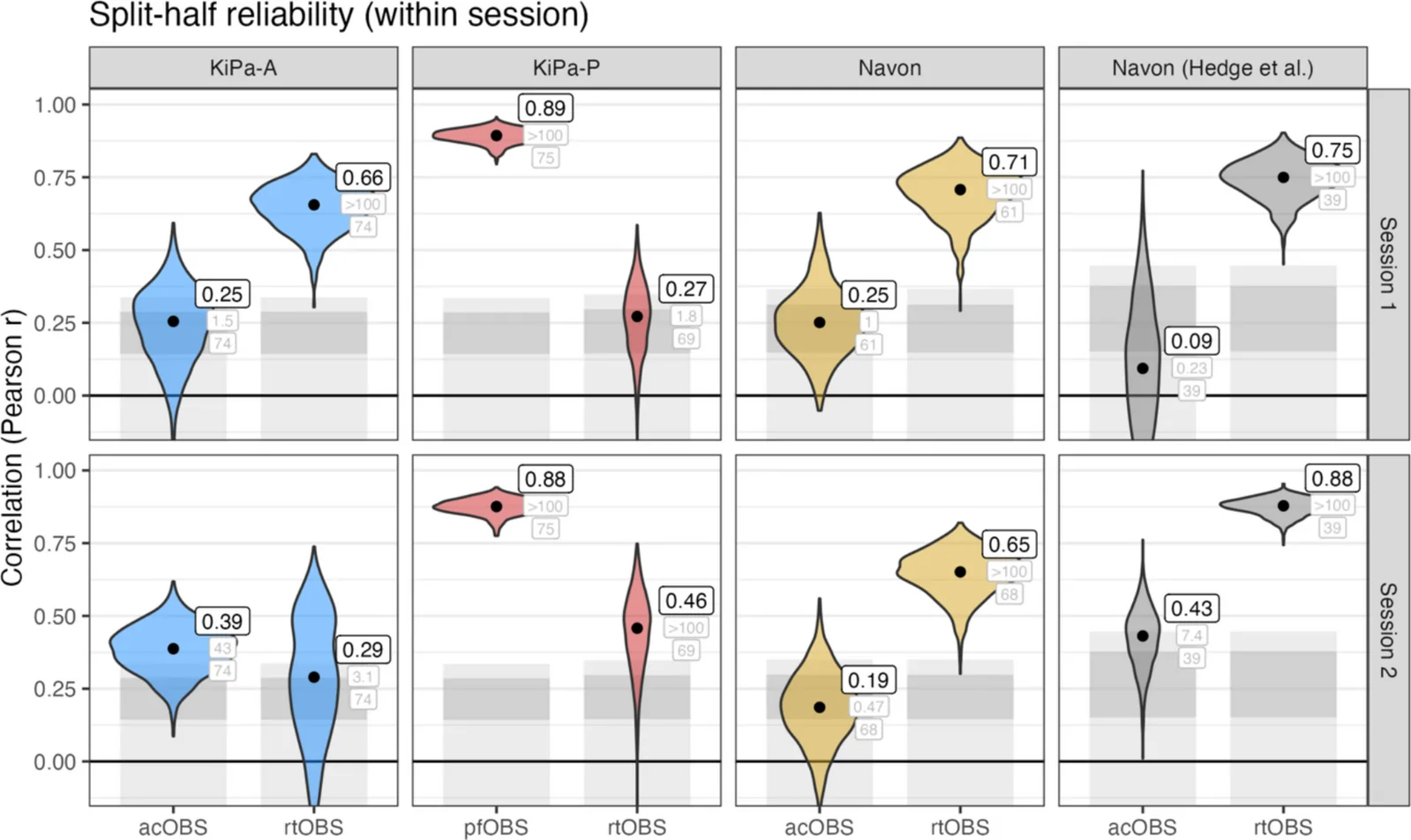
Split-half reliabilities of all task metrics in Session 1 (**top panels**) and Session 2 (**bottom panels**). Each participant’s trials were split into odd and even trials, the scores were recalculated for each half, and the two resulting scores correlated with each other **Data transparency**: Scatter plots for all correlations are shown in Appendix #7 (OSM). Exclusions were performed on raw data only (see Exclusions) **Bootstrapping**: Each violin contains 1,000 bootstrapped correlations and their median. We performed non-parametric bootstrap resampling with replacement (n-out-of-n) **Bayes factors**: Dark grey areas mark the range in which the Pearson’s r values amount to anecdotal evidence for either H0 (towards the lower end) or H1 (towards the upper end). Light grey areas mark the range in which the Pearson’s r values amount to moderate evidence for either H0 (when going towards Pearson r of 0) or H1 (when going towards Pearson r of 1). The white areas mark the range in which the Pearson’s r values amount to strong evidence for H1. For more details on Bayes factors and bootstrapping, see Statistics in the *Methods* section **Terms**: In the figure, KiPa refers to the Kimchi-Palmer task, OBS is the overall bias score, the prefixes ac, rt, and pf stand for accuracy, reaction time, and preference, respectively. For details, see the Metrics section. **Values**: The black value labels on top of the violins indicate the median Pearson’s r of the bootstrapped correlations. The grey labels are the corresponding Bayes factors (based on median Pearson’s r and the number of participants in the correlation) and number of participants in the correlation. For details see Statistics

Split-half reliability is the correlation between scores from odd and even trials within a session. It can give a useful estimate of a metric’s signal-to-noise ratio and thus how meaningful results derived from it are, in particular whether non-correlations are meaningful (one would not expect to observe a correlation between two sets of mostly noise). It is also important to keep in mind though that observed split-half reliabilities are usually an underestimation, because each score is derived from half the number of trials, which exacerbates the impact of random effects. Despite this, we obtain moderate to large split-half reliabilities for several metrics (Fig. 4).

In particular, the split-half reliability of the preference-based OBS in Kimchi-Palmer is strong in both sessions (Pearson’s r ≈ 0.9) and the reaction-time based OBS in Navon is moderately strong (r ≈ 0.7). An open dataset of a two-session Navon task with a comparable number of participants and over six times more trials (Hedge et al., 2018) achieves even higher split-half reliabilities (r ≈ 0.8–0.9). The accuracy-based Navon OBS is only weakly split-half reliable (r ≈ 0.2, Fig. 4)

For Kimchi-Palmer, the two reaction time-based OBS and the accuracy-based OBS show a significantly lower split-half reliabilities (r ≈ 0.3–0.7) than the preference-based OBS and also vary significantly between sessions. The accuracy-based OBS is even weaker (r ≈ 0.3–0.4).

Reaction time and near-ceiling accuracy are generally noisy measures and tend to require a significant number of trials to produce a stable split-half reliability. Our two Kimchi-Palmer subtasks have only 32 trials each, which is further halved during the split-half correlation when correlating one half with the other. Sixteen trials are thus enough for the preference OBS to produce reliable values, but not for the other Kimchi-Palmer metrics.

#### Test-retest reliability

**Fig. 5:**
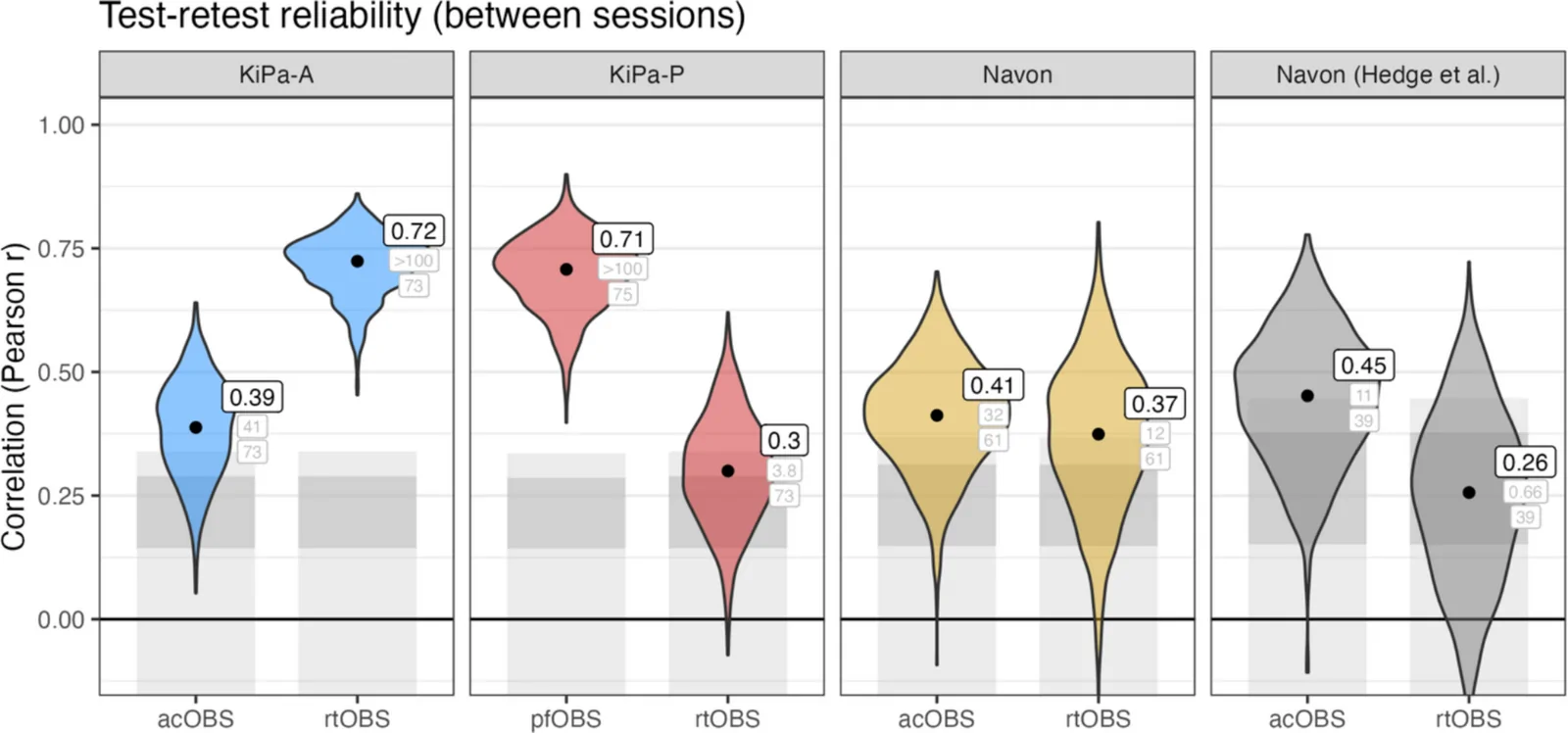
Test-retest reliability correlations of various metrics in the Navon task and the Kimchi-Palmer preference-subtask (KiPa-P) and accuracy-subtask (KiPa-A)

To test the reliability of metrics, i.e., how *trait-like* they are, we performed a Pearson’s test-retest correlation between Session 1 and Session 2 for metrics of each task (Fig. 5).

For the Navon reaction time OBS we found a weak-to-moderate reliability (r ≈ 0.4), while Hedge et al.’s (2018) dataset shows an even lower reliability (r ≈ 0.3), possibly due to differences in task design (smaller stimuli, white-on-black, variable positions on screen) or the lower number of participants. The Navon accuracy OBS, interestingly, shows the same test-retest reliability as the reaction time OBS, despite its much lower split-half reliability (Fig. 5).

In Kimchi-Palmer, the preference-based OBS and the reaction-time-based OBS in the accuracy subtask both have a moderate-to-strong retest reliability (r ≈ 0.7), even though the latter has a much lower split-half reliability (r ≈ 0.4) than the former (r ≈ 0.9). This discrepancy supports the interpretation that the trial number is a limiting factor for the split-half reliability of this metric and possibly other metrics as well. This interpretation is further supported by the fact that the reaction time OBS of the preference task and the accuracy OBS have a comparable reliability between-session (r ≈ 0.3–0.4, test-retest, 1 week apart, Fig. 5) to that within-session (split-half, Fig. 4).

Notably, the Navon reaction time OBS shows a considerably lower retest-reliability (r ≈ 0.4) than the comparable reaction time OBS of the Kimchi-Palmer accuracy subtask (r ≈ 0.7), despite being based on three times as many trials (96 vs. 32) and showing a slightly higher split-half reliability (Fig. 4). These two metrics are superficially identical regarding metric calculation (performance-design, trial level a-priori defined), but the underlying task design and demand differ significantly (see Discussion).

We therefore conclude that the two test-retest reliable metrics (local-global bias in Kimchi-Palmer preference and reaction time OBS in its accuracy subtask) are dominated by an underlying trait effect, that the Navon reaction time OBS is dominated by state effects and noise (very short-term state effects are also indistinguishable from noise when using such tasks), and that the other two Kimchi-Palmer metrics are dominated by trait effects and noise, likely in significant part due to an insufficient trial number in our data.

#### Kimchi-Palmer correlations

**Fig. 6:**
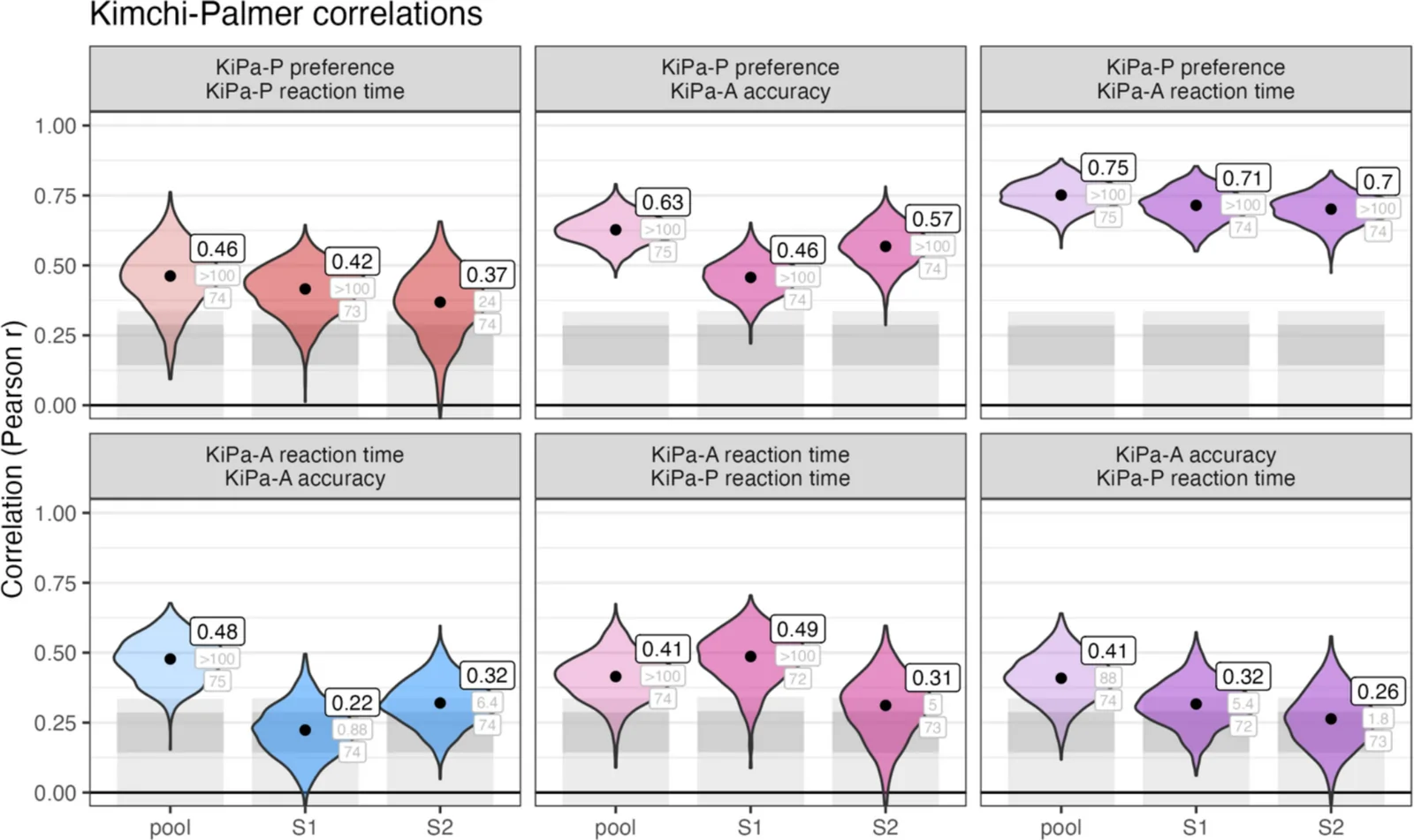
Correlations between the four different Kimchi-Palmer OBS. The left column shows correlations between the two measure-types and within each subtask (each with its respective colour). The middle column shows correlations within measure-type and between subtasks, and the right column shows correlations between measure-types and between subtasks (in pink and purple to visualize the cross-subtask nature of the correlations). The results of “pool” are based on scores from the pooled trials of Session 1 and Session 2 (includes patients whose data are excluded from one session). The results of “pool” are based on scores calculated from the pooled trials of Session 1 and Session 2

To answer our second question of unitarity, we first correlate the four Kimchi-Palmer metrics among themselves, resulting in six pair-wise combinations (Fig. 6). The most inherently interesting correlation is the one between the two high-reliability metrics, the reaction time-based OBS of the accuracy subtask and the preference-based OBS. This correlation is moderately strong (r ≈ 0.7, Fig. 6, top right panel), which is remarkable from a conceptual perspective, considering that it bridges both the subtask (performance-based vs. preference-based) as well as the type of measure (reaction time vs. choice).

The other correlations are weaker, which likely partially reflect their lower split-half reliabilities.

The preference-based OBS is moderately strongly correlated with the accuracy-based OBS (r ≈ 0.5, Fig. 6, top centre panel). The implications of this correlation are as follows: In the accuracy-based subtask, a participant with a global preference tends to find the global match and tends to choose at random when the match is on the local level, suggesting that his brain is either primed to search for global matches or more capable of finding global matches or both.

The reaction-time based OBS of both subtasks are only moderately or weakly correlated with each other (r ≈ 0.4, Fig. 6, bottom centre panel) and with their respective choice-based metric (accuracy or preference) within subtasks (r ≈ 0.2–0.4, Fig. 6, left). Considering the low split-half reliabilities of some of these metrics (as low as r ≈ 0.1), these correlations are nevertheless notable. In particular, it needs to be borne in mind that for strongly globally biased participants, the median local reaction time in the preference subtask may be based on less than a handful of trials (a trial is flagged as global or local by the participant’s preference), which is very low for reaction time data.

Overall, there is a clear trend of positive correlations across subtasks and measure types within the Kimchi-Palmer task.

We also perform the same cross-measure correlation for the Navon task: We compare the reaction time-based OBS with the accuracy-based OBS, and find moderate correlation (r ≈ 0.3–0.4, Appendix #2, Fig. S2 (OSM)) between the two. Given the moderate test-retest reliability of both (r ≈ 0.4) and the weak split-half reliability of the Navon accuracy OBS (r ≈ 0.2), the comparatively strong cross-measure correlation suggests that such a cross-measure may be a general feature of local-global bias tasks, though with varying magnitude.

#### Between-task correlations

**Fig. 7:**
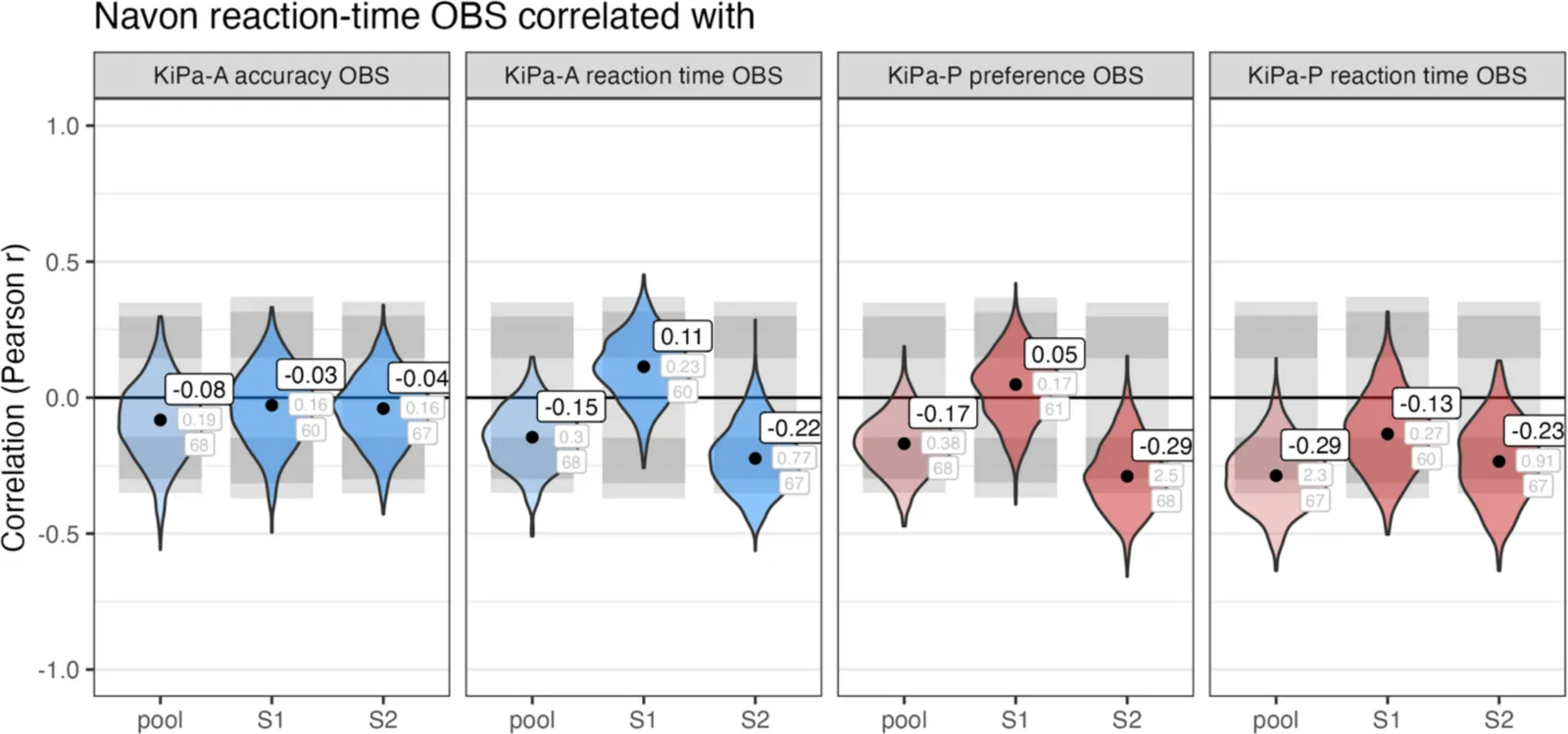
Correlations between Navon reaction time OBS and metrics from both Kimchi-Palmer subtasks (KiPa-A and KiPa-P). See Fig. S8 in Appendix #3 (OSM) for the same correlations but with the Navon accuracy OBS. The only correlations that reach evidence for H1 are for negative Pearson’s r. All positive correlations provide moderate evidence in favour of H0. The results of “pool” are based on scores from the pooled trials of Session 1 and Session 2 (includes patients whose data is excluded from one session)

After establishing the reliability and the internal coherence of both tasks, we finally test the often-implicit assumption of unitary local-global bias by correlating the four Kimchi-Palmer metrics with the reaction-time based Navon OBS (Fig. 7). We find mostly weak correlations hugging a Pearson’s r of 0, with the strongest positive correlation being r ≈ 0.1. All other correlations are weaker or negative, with the strongest correlation reaching r ≈ −0.3. Even without accounting for multiple comparisons, half of the corresponding Bayes factors indicate merely anecdotal evidence for H1 or H0 (all of which for negative correlations) and the other half shows moderate evidence for H0.

The two KiPa metrics that we showed to be most reliable before (the reaction-time based OBS in the accuracy subtask and the preference-based OBS; panels 2 and 3 in Fig. 7) both fit this overall pattern. Notably, they show a clear effect of session, with essentially no correlation in the first session and a negative correlation in the second session (given that they strongly correlate, the two metrics are expected to move in unison). It is not clear whether this reflects a true effect or a spurious correlation. Keep in mind that the Kimchi-Palmer metrics are correlated and the negative correlation trend in Session 2 is therefore not independent but rather due to this underlying within-Kimchi-Palmer correlation.

We also perform the same correlations with the accuracy-based Navon OBS (Appendix #3, Fig. S8 (OSM)) and find a very similar pattern of correlations, most likely reflecting at least in part the moderate correlation between the reaction time OBS and accuracy OBS of Navon that we described in the previous section.

To broaden our perspective to tasks that are not hierarchical compound tasks but still contain a conceptual local-global aspect, we also correlate the field dependence score derived from a Rod-and-Frame task with the metrics of our Kimchi-Palmer and Navon tasks and find no evidence for a correlation (see Results in Appendix #8 (OSM)).

## Discussion

### Interpretation

We find that the reaction time-based and accuracy-based OBS of Navon have a weak-to-moderate retest reliability of r ≈ 0.4, while one metric from each Kimchi-Palmer subtask has a fairly high retest reliability of r ≈ 0.7. These results are broadly in line with similar analyses in previous studies (Dale & Arnell, 2013; Hedge et al., 2018), although we use partially different metrics and a higher number of participants. The high reliability of two Kimchi-Palmer metrics indicates that at least these two effect metrics are predominantly caused by a participant’s bias trait, while Navon metrics are not. As the task design is constant between sessions, the bias variability of Navon must therefore be caused by the remaining third of the three effect-groups that we introduced, namely state effects.

Importantly, these state effects could hypothetically be anything from nearly random states of the neural system at any given moment to very stable cognitive states that could stretch over hours (e.g., mood), days, or even longer periods (e.g., personality disorders or psychiatric disorders). Aside from the source of these effects, it seems that Navon-like tasks require a relatively large number of trials to generate split-half reliable bias scores (i.e., scores that are reliable for the current state), which may be due to the use of reaction time as a measure.

A possibility we did not explore in this study is whether the split-half reliability of the Navon task could be increased by specific interventions, i.e., whether inducing specific cognitive or emotional states might have a differential effect on participants. To our knowledge, such interventions have so far been focused on group-level effects (e.g., Noguchi & Tomoike, 2016).

The relatively good test-retest reliability of the Kimchi-palmer preference-based OBS and reaction time-based OBS of the accuracy subtask shows that that at least for these two metrics, random internal state effects and noise are fairly weak compared to the trait effect. Perhaps, with a higher trial number the other two metrics could rise to a higher reliability as well. In particular, the fact that the accuracy subtask of the Kimchi-Palmer task can produce a decently test-retest reliable reaction time-based metric with roughly 30 trials while the Navon-task struggles to do so (r ≈ 0.4) with roughly 90 trials is to us an indication that the Kimchi-Palmer task is much better suited for individual differences research than the Navon task. On the other hand, while we expect induced state effects (e.g., induced emotions) to be detectable in the Kimchi-Palmer task, the Navon task might be more amenable to them and might be better suited for research on state-bias.

In line with these results, we also find that the correlations between Kimchi-Palmer metrics and Navon metrics (from reaction time-based and choice-based metrics alike) are mostly close to 0 and the resulting Bayes factors mostly provide anecdotal to moderate evidence for the absence of a correlation (Fig. 7). Those few correlations with anecdotal evidence for a correlation are all negative (note, however, that we do not account for multiple testing and there is thus a higher chance to find spurious correlations). Given these findings, one could hypothesize that the two tasks do not capture different aspects of a singular cognitive mechanism but rather entirely different mechanisms, in which case they should not be used interchangeably. A hypothesis that “sensory mechanisms” and “cognitive mechanisms” give rise to different local-global effects was proposed previously (Poirel, Pineau, & Mellet, 2008a, 2008b) and seems worth exploring further.

Importantly, however, an observable lack of correlation does not strictly rule out that the tasks tap into similar or even the same cognitive mechanism – perhaps into different facets or modes of a single mechanism or perhaps different stimuli are simply affected differently by the same mechanism, like wood is smoothened by sanding while metal is roughened by it. Proving or disproving that two observed effects are not associated on the mechanistic level is very difficult and requires a broad convergence of findings across different theoretical aspects of the respective constructs.

Moreover, a possible concern is that our sample size was insufficient for the current goals, since “strong” evidence for the null finding cannot be obtained with our sample size (> 60 participants) and even correlations closing in on 0 stay below a 10:1 likelihood ratio in favour of H0. However, even assuming that anecdotal moderate evidence for the null finding was insufficient for our purposes, we think that if biases in the two tasks are driven by the same mechanism, this should still appear in our data to a meaningful degree. Detecting an arbitrary strong correlation of r = 0.5 with 80% power (using alpha = 0.025, one-tailed) would require *N* = 35 participants (Hulley et al., 2013). The sample used in the current study is substantially larger, and yet we do not find any clear correlation between Kimchi-Palmer and Navon when using the OBS metric for both tasks, neither for the reaction time of the accuracy-based Kimchi-Palmer nor for the preference in the preference-based Kimchi-Palmer.

Another possibility for not observing a correlation is that measurement noise precludes one from detecting a true correlation. In our case, this seems unlikely, given the mostly reasonably high split-half reliability (note that split-half reliability underestimates true reliability since it halves the number of trials on which a score is based; see details in *Results*). A correlation of, for example, r = 0.5 should still be detectable with the observed split-half reliabilities (Nicewander, 2018; Spearman, 1904).

Considering the above, we conclude that the effects in Navon and Kimchi-Palmer, while being local-global effects, do not represent a *unitary* local global effect. With this paper we want to invigorate the debate around the conceptual assumptions of local-global research as we think the lack of an observable correlation is remarkable for a field that uses the broad and unitary terms “local global bias” or “global advantage” so ubiquitously.

Previous studies have examined the nature of local-global stimuli as well (Poirel, Pineau, & Mellet, 2008a, 2008b) or even correlated the Navon and Kimchi-Palmer tasks directly (Dale & Arnell, 2013; Pletzer et al., 2017), but there seems to be a wide gap between attention to this matter and the significant conceptual and theoretical implications that this finding has for the field.

At this point we do have to note that while we come to the same general conclusion as Dale and Arnell (2013) and Pletzer et al. (2017), the strength of their empirical findings is limited or even invalidated by their choice of metrics: Dale and Arnell used the preference-based OBS for the Kimchi-Palmer task and the global-to-local interference score (GIS) for Navon (Dale & Arnell, 2013). Since the GIS is not in itself a local-global bias metric (Schweigkofler et al., 2025), correlating it with an OBS – which *is* a local-global bias metric – is conceptually not very meaningful. Pletzer et al., on the other hand, used only the reaction time-based OBS from the Kimchi-Palmer preference-subtask (Pletzer et al., 2017), which is – as we show – not very reliable. In our opinion, the metrics is interesting but not theoretically and empirically established enough to allow for strong interpretations. Thus, while conclusions about the missing link between the Navon and Kimchi-Palmer tasks already existed in the literature, the presented results were based on single and questionable metrics and thus until now insufficient to meaningfully support the conclusions drawn from them.

More recently, other variations of hierarchical compound figure tasks were found to be not correlated with each other as well (Lacko et al., 2023), strengthening the impression of a highly facetted and fragmented local-global bias effect space.

We also find that our new version of the Rod-and-Frame field dependence (which is not yet cross-validated with traditional versions) is not correlated with the Kimchi-Palmer task or Navon task (see Results in Appendix #8 (OSM)). Two previous studies that correlated field dependence (derived from Embedded Figure Tasks and not the Rod-and-Frame task) against a different hierarchical compound figure tasks found no correlation (Booth, 2006; Chamberlain et al., 2017), while a third found a medium strong correlation (Poirel, Pineau, Jobard, et al., 2008a, 2008b). Due to the numerous and significant differences between task designs, any conclusions drawn from these cross-construct correlations is speculative, but the correlation in the latter study suggests that with a deeper and more comprehensive understanding of the constructs involved, meaningful insights may be gained from such correlations across task types.

Lastly, we find relatively strong correlations between the two subtasks of the Kimchi-Palmer task (Fig. 6), suggesting that the cognitive processes responsible for speed, accuracy, and preference might be connected, at least within a particular task design. We can thus infer that the task type – preference or performance – likely does not explain the differences between Navon and Kimchi-Palmer, as was hypothesised previously (Goodhew & Edwards, 2019). Again, however, these findings should not be generalised prematurely to other design variations.

Taken together, our results strengthen previous impressions that local-global bias is not a unitary construct and that observable local-global bias effects are strongly influenced by the specific task design, even when their basic underlying logic seems similar.

#### Task differences and future research

The first limitation of our experiment – and the entire field of local-global bias – is thus that we cannot generalize from one task. Secondly, the Navon task and the Kimchi-Palmer task differ in so many aspects that we cannot point to any specific design characteristic as being responsible for their different behavioral outcomes. We therefore want to highlight three major differences that can serve as a starting point for further investigation.

**Attentional focus**: In the Navon task, participants’ focus is directed towards the relevant level, while in the Kimchi-Palmer task attention is divided between both levels. This could potentially elicit a different bias mechanism, as attentional focus could overwrite a stable, but weak “default” focus on a certain level that is captured by Kimchi-Palmer, but not Navon.

**Cognitive load**: A second major difference is the much higher cognitive load in Kimchi-Palmer compared to Navon. An additionally increased cognitive load in Kimchi-Palmer was found to reduce the global bias for unlimited viewing times (Hoar & Linnell, 2013), but in Navon it was found that cognitive load increases the global interference bias by increasing global-to-local interference and decreasing local-to-global interference (Ahmed & de Fockert, 2012). Note that both findings refer to group-level shifts, not individual correlations.

**Memory**: An even greater difference between Navon and Kimchi-Palmer is that Kimchi-Palmer requires working memory, while Navon does not. It could therefore be that Kimchi-Palmer does not measure a perception bias, but rather a memory bias. Possibly, an unstable and easily manipulable perception bias exists independently from a more stable visual working memory bias.

Further potentially relevant differences between our Navon and Kimchi-Palmer are the visual features, as letters might be processed differently from shapes, as well as vagueness of the instructions of the Kimchi-Palmer task (see Tasks in *Methods*) that might prompt some participants to approach the task differently than others, or the type of response that is required (report figure in Kimchi-Palmer, report feature in Navon) that might again have a differential impact on participants’ strategies.

These design differences may serve as a starting point for a hopefully comprehensive examination of local-global task designs. Without a better understanding of how each aspect of the task design influences the bias effects, local-global bias research would go on to be less precise, less efficient, and less impactful than it can be.

## Data Availability

Formatted data are available in the data repository of our study (osf.io/z39j2/) in the logloRxiv (local-global bias archive) as well as in the project repository (osf.io/cm3nh/).

## Footnotes

1 If a participant sample contains subgroups with widely different group-based trait effects (i.e., a wide spread), the retest-reliability is higher (for the same within-participant variation). While it is difficult to draw a clear line between what counts as an individual difference and what counts as group difference, we thought it prudent to aim for a homogenous sample for our individual-differences study. As we are unaware of any stark group differences that would be meaningful for a general European population, we assumed that a young UK group that can understand our instructions fluently is sufficiently representative for our purposes.

2 On a screen pictures are displayed in pixels, not in millimetres and every participant’s screen had a different resolution. We used the credit-card-size-check to find out the resolution of their screen to adjust the display of the figures in pixel (scale factor). Due to this, some inaccuracies on the participants side can occur. Four participants had a different scale factor in Session 1 and Session 2 (more than twice the size or less than half), possibly due to using a different device. Excluding them did not have a relevant effect on the results.

## Code availability

The code and materials to replicate the experiment and format the raw data are available in the data repository (osf.io/z39j2/). The code to replicate the analysis is available in the project repository (osf.io/cm3nh/).

## Declarations

### Open practices statement

The experiment code, raw trial-level data, pre-processing code, and pre-processed trial-level data are stored in the Open Science Framework (OSF)-based logloRepo (local-global bias repository, osf.io/z39j2). The analysis code and pre-processed trial-level data are available in the project repository of this article (osf.io/cm3nh). The analysis was not preregistered.
